# Neural network assisted annotation and analysis tool to study *in-vivo* foveolar cone photoreceptor topography

**DOI:** 10.1038/s41598-025-08028-9

**Published:** 2025-07-04

**Authors:** Aleksandr Gutnikov, Patrick Hähn-Schumacher, Julius Ameln, Shekoufeh Gorgi Zadeh, Thomas Schultz, Wolf Harmening

**Affiliations:** 1 Department of Ophthalmology, University Hospital Bonn, Bonn, 53127 Germany; 2 b-it and Computer Science Department, University of Bonn, Bonn, 53115 Germany; 3 Lamarr Institute for Machine Learning and Artificial Intelligence,; 4 Present Address: Luxembourg Centre for Systems Biomedicine, University of Luxembourg, Belvaux, Luxembourg

**Keywords:** AOSLO, Neural networks, FCN, Confocal imaging, Retinal diseases, Perception, Adaptive optics, Computer science, Software

## Abstract

The foveola, the central region of the human retina, plays a crucial role in sharp color vision and is challenging to study due to its unique anatomy and technical limitations in imaging. We present ConeMapper, an open-source MATLAB software that integrates a fully convolutional neural network (FCN) for the automatic detection and analysis of cone photoreceptors in confocal adaptive optics scanning light ophthalmoscopy (AOSLO) images of the foveal center. The FCN was trained on a dataset of 49 healthy retinas and showed improved performance over previously published neural networks, particularly in the central fovea, achieving an $F_1$ score of 0.9769 across the validation set, critically reducing analysis time. In addition to automatic cone detection, ConeMapper provides efficient manual annotation tools, visualizations and topographical analysis, offering users detailed metrics for further analysis. ConeMapper is freely available, with ongoing development aimed at enhancing functionality and adaptability to different retinal imaging modalities.

## Introduction

The foveola, the central 1-degree diameter of the human retina, is a sought-after study target for ophthalmology, vision science, and the neurosciences^1–4^. This is in part due to its key role for human vision, being the retinal area for sharp color vision^5–7^, and at the same time, the historical difficulty to study its delicate anatomy with both *ex-vivo*^8^ and *in-vivo* approaches^9,10^. With the recent refinement of high-resolution retinal imaging techniques equipped with adaptive optics, researchers can directly observe the mosaic of the light-sensitive cone photoreceptors and their outer segments in the foveola of the living eye^11–14^, and study its functional role for human vision in health and disease^15–18^.

*In-vivo* imaging and topographical analysis of the foveal center remain challenging, even under optimal imaging conditions—such as minimized ocular aberrations, clear optical media and minimal eye movements. This is because the outer segments of foveolar cones are long and thin, thinner than anywhere else in the retina^19,20^. Their minimal diameter, typically less than 2 ${\upmu }$m, approaches the lateral resolution limit governed by the laws of diffraction of light focused in a human eye^21^. Combined with their orderly arrangement, with an almost crystalline packing geometry and a steep density gradient towards a central peak^22,23^, imaging with insufficient lateral resolution produces spurious patterns. Such patterns, similar in appearance to cellular structure, may mask the true underlying mosaic and hinders image interpretation^24^. Moreover, the outer segment’s wave-guiding properties cause a natural variation in reflectivity in confocal images which can make individual cells disappear from the mosaic^25–28^.

In the past years, image analysis and automatic cell annotation techniques have been put forward to quantify rod and cone photoreceptor cells in confocal and non-confocal retinal images, as they are produced by modalities such as adaptive optics scanning light ophthalmoscopy (AOSLO). Such solutions span a variety of approaches, including image histogram analysis^29^, and multiscale modelling and normalized cross-correlation^30,31^. More recently, neural networks (NNs) have been developed to generalize cell identification from a manually labelled training data set^32–36^. While NNs have been shown to work reliably in non-foveal areas where cell density is relatively constant, they have not been trained nor applied for foveolar analysis. The gold standard for foveolar image annotation thus remains manual labeling by an expert image reader^3,17,37^. Manual image annotation is, however, extremely time-consuming, prone to error, and highly dependent of internal annotation criteria employed by the reader in the interpretation of images that are affected by the challenges described above^38,39^. While individual research labs have developed their own analysis strategies that can lead to difficulties when data is shared between sites, there is currently no open-source solution for researchers to efficiently annotate and analyze foveolar AOSLO imagery.

Here, we present an open-source software package that integrates an improved state-of-the-art neural network with an approachable user interface for fast, reliable automatic detection, efficient manual relabeling and basic topographical analysis of the human foveolar cone mosaic.

## Methods

A MATLAB application (“ConeMapper”) was developed that integrates a fully convolutional neural network (FCN) with additional annotation, visualization and analysis functionality. ConeMapper was developed in MATLAB 2022b App Designer. The FCN and post-processing steps were developed in Python with PyTorch library and are called as external Python scripts during runtime of the Matlab app. To gauge our FCN results, two published neural networks were trained and evaluated with identical data sets. For the Cunefare CNN, the MatConvNet 1.0-beta25 library was used.

### Data set

**Figure 1:**
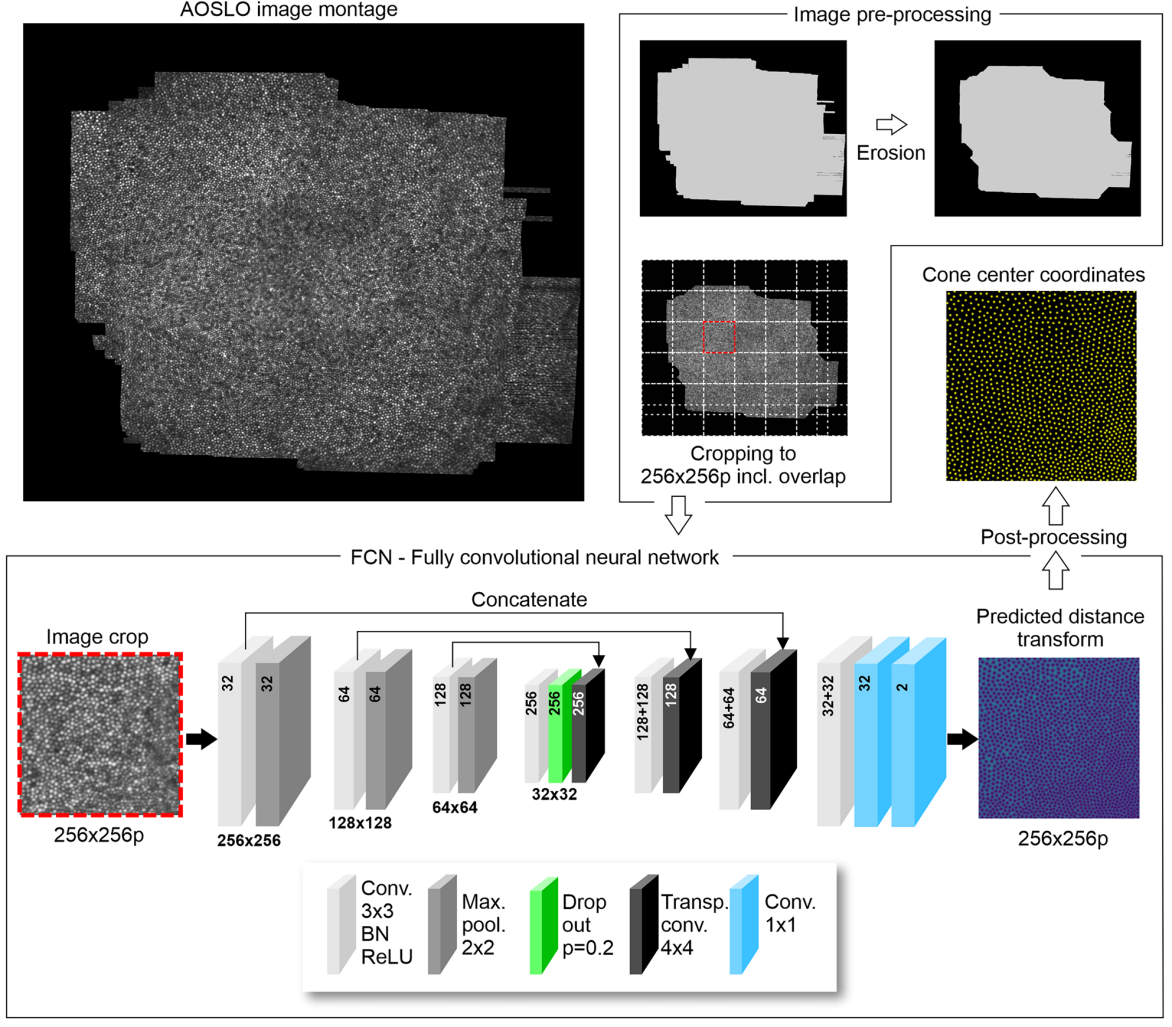
Image data workflow during automatic cone detection. AOSLO image montages were eroded and cropped (top) to serve as image data for a fully convolutional network (FCN, bottom). From the resulting predicted distance transform, cone center locations were derived for each crop and stitched back into the full montage.

The dataset of confocal detector AOSLO images has been acquired for another study focusing on foveal cone topography^40^. It consisted of intensity image montages of 49 healthy retinas (ages, average: 25 years, range: 10-44 years), each covering an approximate 2-degree diameter of the retina centered on the foveola (see Figure 1, top left). The AOSLO system was described in more detail previously^41,42^. The digital resolution was 600 pixels per degree of visual angle. Imaging wavelengths were 711, 788, or 840 nm. Each montage was composed of 9 summed and normalized images which were computed from 10-second AOSLO video recordings. Individual video frames were stabilized offline using a refined strip-based registration technique based on an earlier published algorithm^43^, and then pixel-wise summed and normalized. This process eliminated uncorrelated detector noise. Individual sumnormed images were automatically aligned using software previously described^44^ and manually blended in Corel PhotoPaint (v21.0.0.593, Corel Corporation, Ottawa, Canada) to create a continuous image montage. In each montage, cone center locations were annotated using then available annotation tools and by manual correction.

For network training, the dataset was randomly split into a training set of 39 montages and a test set of 10 montages (80:20 split). Each montage was cropped into randomly chosen, overlapping 256 patches of size 256 $\times$ 256 pixels and processed with their corresponding labels. Before cropping, montage borders were eroded to counteract image artifacts such as noise and striping. We used a binary structuring element representing a von Neumann neighbourhood, which took only directly adjacent pixels into account. Due to the limited data availability, only 64 erosion iterations were performed on each montage, which eliminated most artifacts while keeping as much image content as possible (Fig. 1, top right). Erosion was also employed during recognition as a pre-processing step, though the number of iterations was limited to two. This is because each iteration removes part of the border information. During the training phase, it is crucial to retain as much clear data as possible, whereas in the recognition phase, we aim to utilize as much image data as we can. Two iterations of erosion were sufficient to eliminate the most significant artifacts at the borders.

### Fully convolutional network

The network architecture represented a modified version of U-Net^45^ and closely resembled a fully convolutional network proposed earlier^34^. It consisted of an encoder-decoder like structure with no fully connected layers after the decoder. A number of modifications to the earlier implementation were employed, such as different label representations, input sizes and number of filters, mainly to satisfy an increased network capacity (Figure 1). Our solution employed a single forward pass to process an image.

The encoder consisted of multiple blocks at a given resolution, which are connected by max pooling layers decreasing the resolution. Each block consisted of k sub-blocks, each containing a 3 $\times$ 3 convolutional layer with zero-padding, a batch normalization layer and a ReLU layer. The number of filters is increased by a factor of 2 within each block. Best performances were obtained using k = 2 sub-blocks at each resolution. As in the original implementation, we chose 3 decreases in resolution by pooling which was able to capture all cone sizes present in the images.

In contrast to the original U-Net, Hamwood et al.^34^ inserted a dropout layer at the lowest resolution between the encoder part and decoder part of the neural network due to the need for regularization when training with a limited amount of data. However, their chosen dropout probability of p = 0.5 led to worse results than a more lenient value of p = 0.2 with our available data.

The decoder consisted of multiple blocks at a given resolution as well. The output of a previous resolution block is upscaled to doubled resolution using transposed convolution with a kernel size of 4 $\times$ 4, a stride of 2 $\times$ 2 and zero-padding of 1 $\times$ 1, following the reference implementation of Hamwood et al.^34^. In addition to the upscaled feature maps, the output of the encoder block of the same resolution is concatenated using skip connections before being processed by the respective block. Skip connections have proved to be beneficial in deep neural networks when reconstructing after decreasing the resolution to not lose structural information^46^. Like the encoder blocks, the decoder blocks consist of k = 2 sub-blocks, each with a 3 $\times$ 3 convolutional layer with zero-padding, a batch normalization layer and a ReLU layer. However, the number of filters is decreased by the decoder block by a factor of 2.

While U-Net has been originally designed for segmentation^45^, we modified it to perform regression due to our chosen label representation of a non-negative distance transform instead of class probabilities. We replace the last softmax layer of the neural network with a 1 $\times$ 1 convolutional layer. Hence, we receive a single channel 256 $\times$ 256 network output of a predicted distance transform.

### Post-processing

Finally, to localize cones from the FCN result, we implemented the same approach suggested by Cunefare et al.^32^, by thresholding and determining local maxima in the NN probability map. To further minimize possible manual work after automatic cone detection, a regularity-aware particle system (RAPS) was implemented. This attempts to take advantage of the observed regularity in the GT cone locations to shift cones into place. In the particle system, each cone holds internal and external energy that dictates its location relative to the surround. The whole energy of the system is: $E = (1.0 - \alpha ) \times E_{ext} + \frac{\alpha }{2} \times E_{int}$. Here, $\alpha$ is a control parameter. The external term $E_{ext}$ imposes the environmental information given by the predicted distance transform *D* of the retinal image onto the particles: $E_{ext} = \sum _{p \in P}D(p)$. $E_{int}$ are internal forces that imposes the regularity of the cone mosaic onto the particles by modeling the influence *I* of neighboring particles. Each particle $p \in P$ contributes to the internal term by its internal energy, which is the sum of all influences from neighboring particles $n \in N(P)$: $E_{int} = \sum _{p \in P}\sum _{n \in N(P)}I(p,n)$. Optimal cone locations were then found by minimizing the total energy, E, of the system.

### Validation

For validation, an additional data set of 12 image montages was employed that was not used during NN training/testing. The validation data set was obtained in the same way as described earlier. To quantify and compare the performance of our FCN, the automatically found cone locations were matched one-to-one to the ground truth (GT) coordinates which were found by manual labeling of an expert image reader. An automatically found cone was considered a true positive (TP) if it was located within some distance *d* of the GT coordinates. The value *d* was found empirically and set to 0.35 of the minimal distance between two cones across all the images. Automatically detected cones that were not matched to a GT cone were considered false positives (FP), and GT locations that did not have a matching automatically detected cone were considered false negatives (FN). If a GT cone was matched to more than one automatically marked cone, only the one with the smallest distance was considered a TP, and the remaining were considered FP. Finally, we were comparing the cones only in image boundaries formed by GT sets. Automatically detected cones outside of that boundary were excluded from analysis. From TP and FP, the true positive rate (TPR), false detection rate (FDR) and $F_1$ score ($F_1$) were computed by,

$$\begin{aligned} TPR= & TP / (TP + FN), \\ FDR= & FP / (TP + FP), \\ F_1= & 2 * TP / ( 2 * TP + FP + FN). \end{aligned}$$

Because the seemingly subtle differences in such metrics may be sometimes hard to translate into real-world impact, we added an approximate estimation of correction time needed for a manual re-labelling step, i.e. how much time is required to fix the errors in the NN annotation by a human image reader. This approximation is based on the assumption that an expert reader requires 3 seconds to fix one cone position manually, either by adding a missing FN, or by removing a FP.

To evaluate the RAPS, an additional metric was used. The Chamfer Distance is defined as

$$\begin{aligned} Cham\!f\!er Distance = \frac{1}{|C|} \sum _{c \in C} d_p(c) \end{aligned}$$

where *C* denotes the set of all ground-truth cone locations, P is the set of predicted cone locations and $d_p(c)$ represents the minimum distance between a ground-truth cone location and any predicted cone location.

### Cone density metrics

A commonly used quantitative metric for analyzing photoreceptor mosaics is cone density, defined as the number of cones per unit area. Three different ways of density calculation were implemented in ConeMapper:

1. Voronoi: A map of two-dimensional cone density was computed based on the combined area of the Voronoi cells of the nearest 150 cones to any given point in the map. Vicinity is based on Euclidean distance between points. This method was suggested by Reiniger et al.^17^.
2. ICD: Density is computed at each cone location from the individual average inter cone distance (ICD) and assuming perfect hexagonal packing, by $density = 1 / HexArea; HexArea = ICD^2 \times \sqrt{3} / 2$. ICD for a given cone was computed as the average distance to all its neighbours. Neighbouring cones are all those that share a common edge with the given cone in the Voronoi diagram. For every point in the map which is not a cone location, density is interpolated by using MATLAB function *scatteredInterpolant* with nearest neighbour method.
3. Yellot’s ring: Density is computed based on the spatial frequency content of the image, found by analysis of symmetrical patterns in the Fourier transformed image. This approach does not require annotation. We implemented an algorithm developed and described by Cooper et al.^31^.

One of the features of ConeMapper is a density z-score map, visualizing the difference of a current density map to the group average. For this, group average density maps based on the 49 full montage images were computed for left and right eye separately. The z-score value, z, at each map point is then calculated as,

$$\begin{aligned} z = (x-\mu ) / \sigma \end{aligned}$$

where *x* is the density value at given point, $\mu$ is the average density across all eyes at given point, and $\sigma$ is the density standard deviation across all eyes at given point.

Further, derived from such two-dimensional density maps, density profiles were computed along either horizontal or vertical meridians or as radial averages, in each case centered on the cone density centroid (CDC). The CDC was found in each density map as the weighted centroid of all density values within the contour of the 20% highest density values^17^. Consistently, retinal eccentricity was defined as distance from the CDC throughout all analysis.

### Data and software availability

Image datasets used for training, testing and validation are available at https://doi.org/10.17632/r87cvk4mp8.1. The complete software package of ConeMapper, including example data sets, documentation and roadmap, is available at https://github.com/ukb-aoslo/ConeMapper.

## Results

### Automatic foveolar cone detection

To evaluate automatic annotation performance of our software, key performance metrics were computed for a validation data set and compared to the performance of two published neural networks (Hamwood FCN^34^, Cunefare CNN^32^), and to a conventional fast peak find algorithm (FPF, Fast 2d Peak Finder MATLAB File Exchange) ^47^on the same set.

Across all montages from the validation data set, the two NNs and our solution show similar performance, with our solution performing best, on average (Table 1). As expected, the FPF algorithm does not work well for the challenging task of cone identification in the foveal center. The average $F_1$ Scores across the validation set were 0.8723 for FPF, 0.9517 for Cunefare CNN, 0.9449 for Hamwood FCN, and 0.9769 for our solution. Individual metrics computed for each montage are presented in the Supplemental Information. In some cases, all NNs perform similarly, in others, differences can be quite drastic (compare supplemental Tables S1, S2, S3).

**Table 1:** Comparative performance metrics for automatic foveolar cone detection. TPR: true positive rate, FDR: false detection rate, $F_1$: $F_1$ score. Except for FDR and correction time, higher values indicate better performance.

	FastPeakFinder	Cunefare	Hamwood	Our FCN
Avg TPR	0.9531	0.9484	0.9197	0.9778
Avg FDR	0.1946	0.0447	0.0278	0.0238
Avg $F_1$	0.8723	0.9517	0.9449	0.9769
Avg TPR (0.3 deg)	0.9597	0.9848	0.9681	0.9931
Avg FDR (0.3 deg)	0.4681	0.0289	0.0080	0.0101
Avg $F_1$ (0.3 deg)	0.6799	0.9779	0.9799	0.9915
Median Correction Time (min)	350	117	141	45

**Figure 2:**
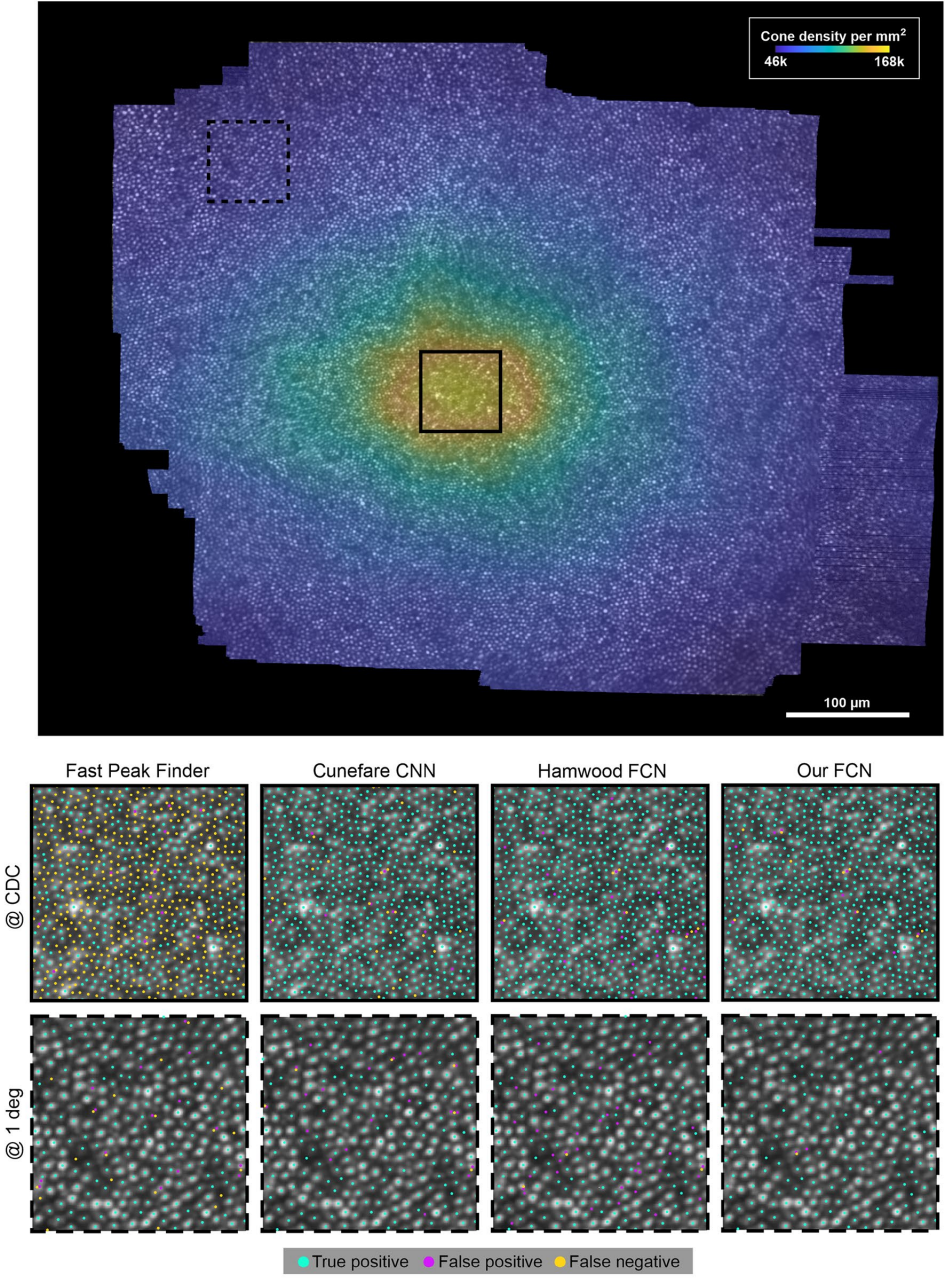
Automatic cone detection quality. Top: GT density map of eye BAK1011R. Density is indicated by color. The squares show the regions presented at higher magnification in the bottom panel, at the CDC (solid line), and at 1-deg eccentricity (dashed line). Box size is 64 ${\upmu }$m. Bottom: Detection results from the Fast peak find algorithm, Cunefare CNN, Hamwood FCN, and our FCN. TP, FP and FN are indicated by colors. All neural networks were trained on the same data set.

Because of the steep gradient in cone density towards the foveal center and the associated rapid change in structural appearance (Figure 2), we additionally compared the performance metrics for the bounding box within 0.3 deg radius from ground truth CDC location (Tables S4, S5, S6). Results show that our solution excels especially in this region, with mean $F_1$ scores of 0.6799 for FPF, 0.9779 for Cunefare CNN, 0.9799 for Hamwood FCN, and 0.9915 for our solution. Inclusion of the regularity-aware particle system (RAPS) did improve cone localization relative to the FCN result slightly. The Chamfer distance was 0.602 without and 0.557 with RAPS. However, the RAPS had no impact on $F_1$ scores, because it did not add or remove cones.

On our test-system running Windows 11 and Matlab R2022b with a Intel Core i5 11600K CPU and NVIDIA RTX3070 graphics card with 8Gb of VRAM, average recognition time of our FCN was 141 seconds per montage without the RAPS and 177 seconds with RAPS.

When the performance metrics were translated to the real-world problem of manual re-labelling after automatic detection, it became clear that our solution outperformed the current best solution by more than 2.5-fold. The median estimated correction time of our FCN was 45 minutes, presenting a significant advantage over median correction times of 141 minutes for Hamwood FCN, 117 minutes for Cunefare CNN, and 350 minutes for FPF (Table S7).

### Cone density maps and profiles

**Figure 3:**
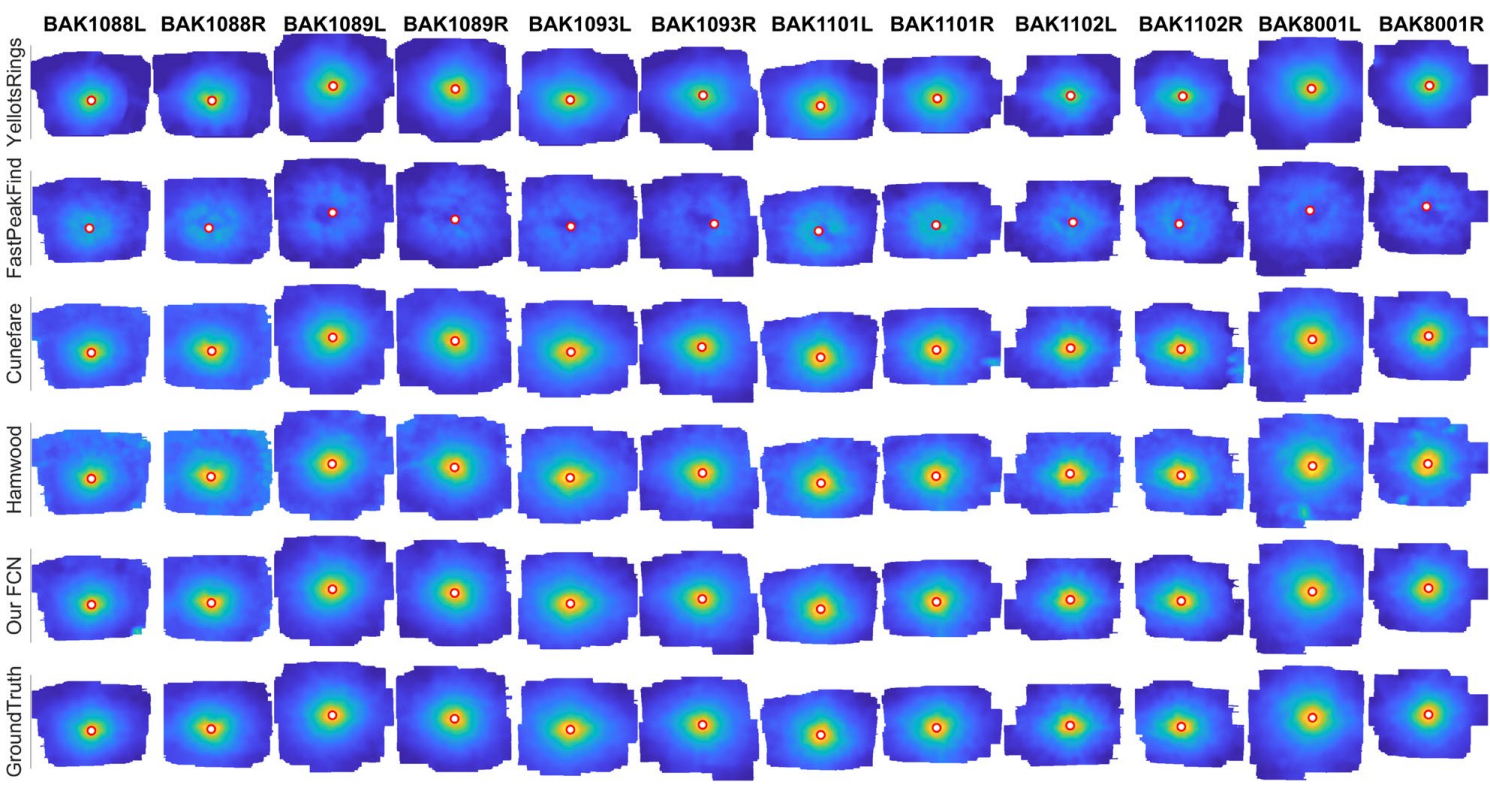
Density maps of the validation data set. The red-white marker in the center represents the CDC. Each column represents one montage, rows are the different recognition algorithms results. The bottom row is GT. Colors are cone density, normalized for GT density in each eye.

**Figure 4:**
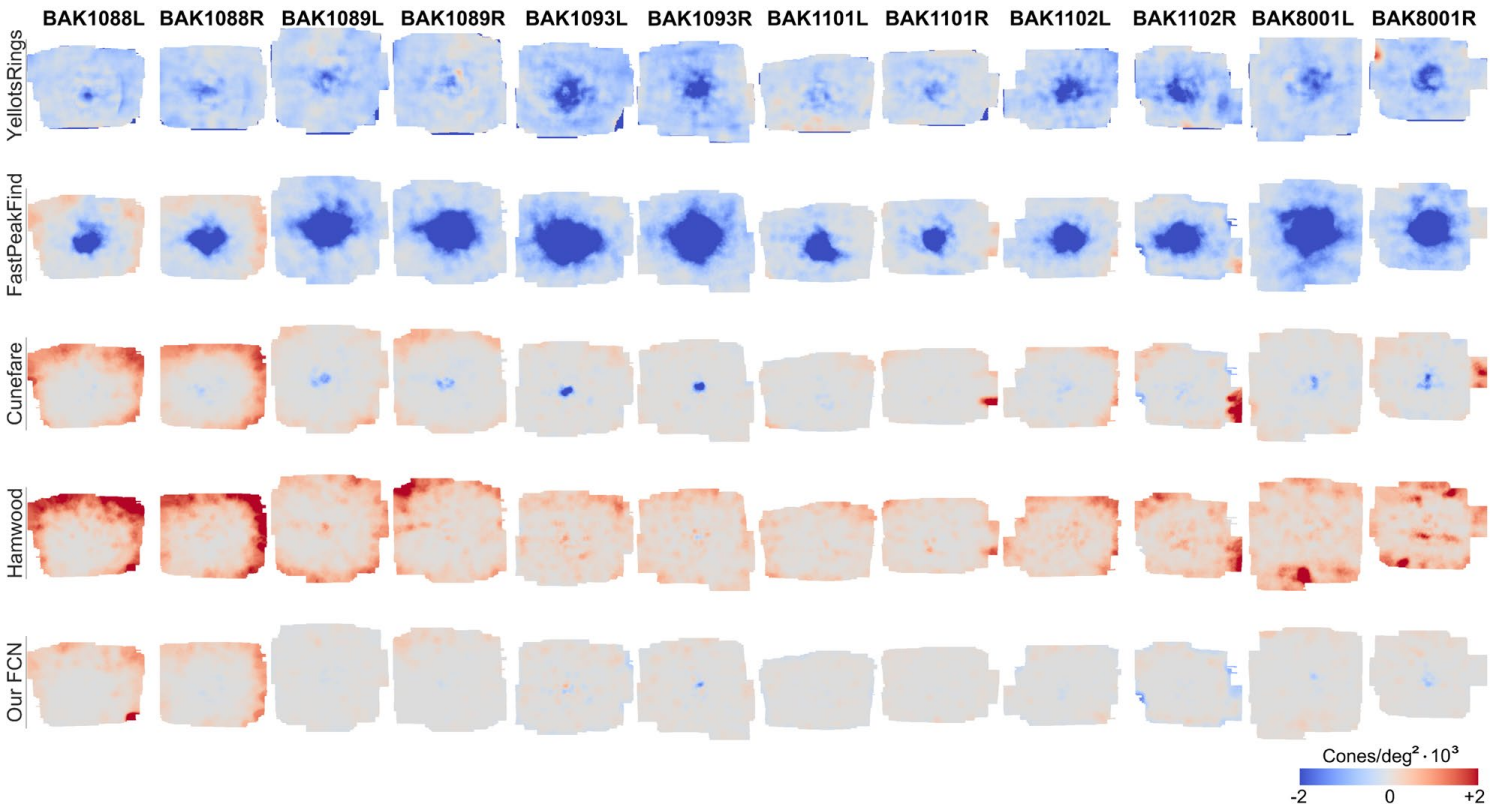
Density difference from GT of the validation set. Red color indicates overestimation, blue is underestimation. No difference is gray.

**Figure 5:**
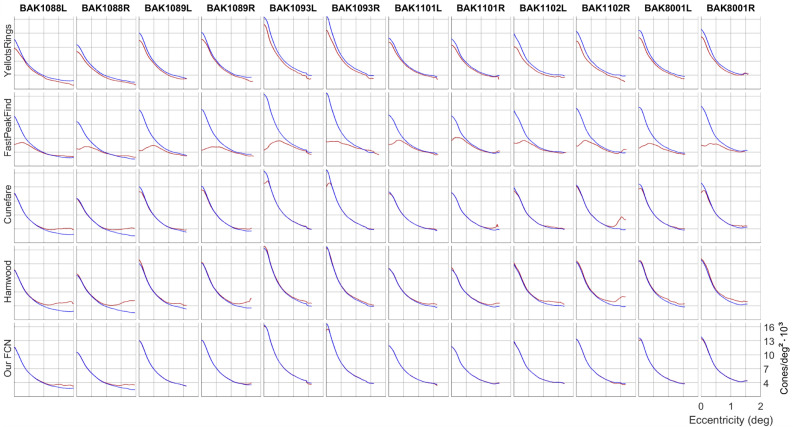
Radially averaged density profiles of the validation set. Red lines are the profile based on the given recognition algorithm. The blue lines are GT profiles.

The uncorrected two-dimensional density maps and density radial averages were also compared to GT data. The comparison in Figures 3, 5 confirms that our solution outperforms the current solutions, especially in the foveal center. We also compared density maps resulting from the spatial frequency image analysis^31^ to GT, because this method is independent of annotation quality. Density difference maps (Figure 4) better highlight the typical problematic regions for each approach. Overall, the typical problematic regions were the foveal center (< 0.2 deg radii from CDC), because of light interference artifacts, and above 1 deg radii from CDC, due to border artifacts and the increased occurrence of rod photoreceptors that are similar in appearance as the smallest cones.

The density radial profiles in Figure 5 show the deviation from GT of each method. It is clearly visible that most of the methods struggled close to the CDC and at radii>1 degree away from CDC. Our FCN performs better at the CDC in most eyes (but compare data for BAK1093R). Interestingly, the Yellot’s Ring approach systematically underestimated density in all cases.

### ConeMapper annotation, visualization and analysis features

As we have seen, automatic cone detection was improved, but still leaves us with undetected or falsely labeled cones. On average, this amounted to about 900 errors in a 2-degree foveal montage, and to about 40 errors in a central zone (0.3 deg radii around CDC). In a newly acquired image, ground truth data is not available, of course. We have thus equipped the ConeMapper software with tools that make the manual correction process as easy and efficient as possible. For this, an intuitive annotation interface, several visualization options and basic analysis features were developed. We briefly describe the main features here. For complete documentation and more examples, the reader is referred to the help section of our app.

**Figure 6:**
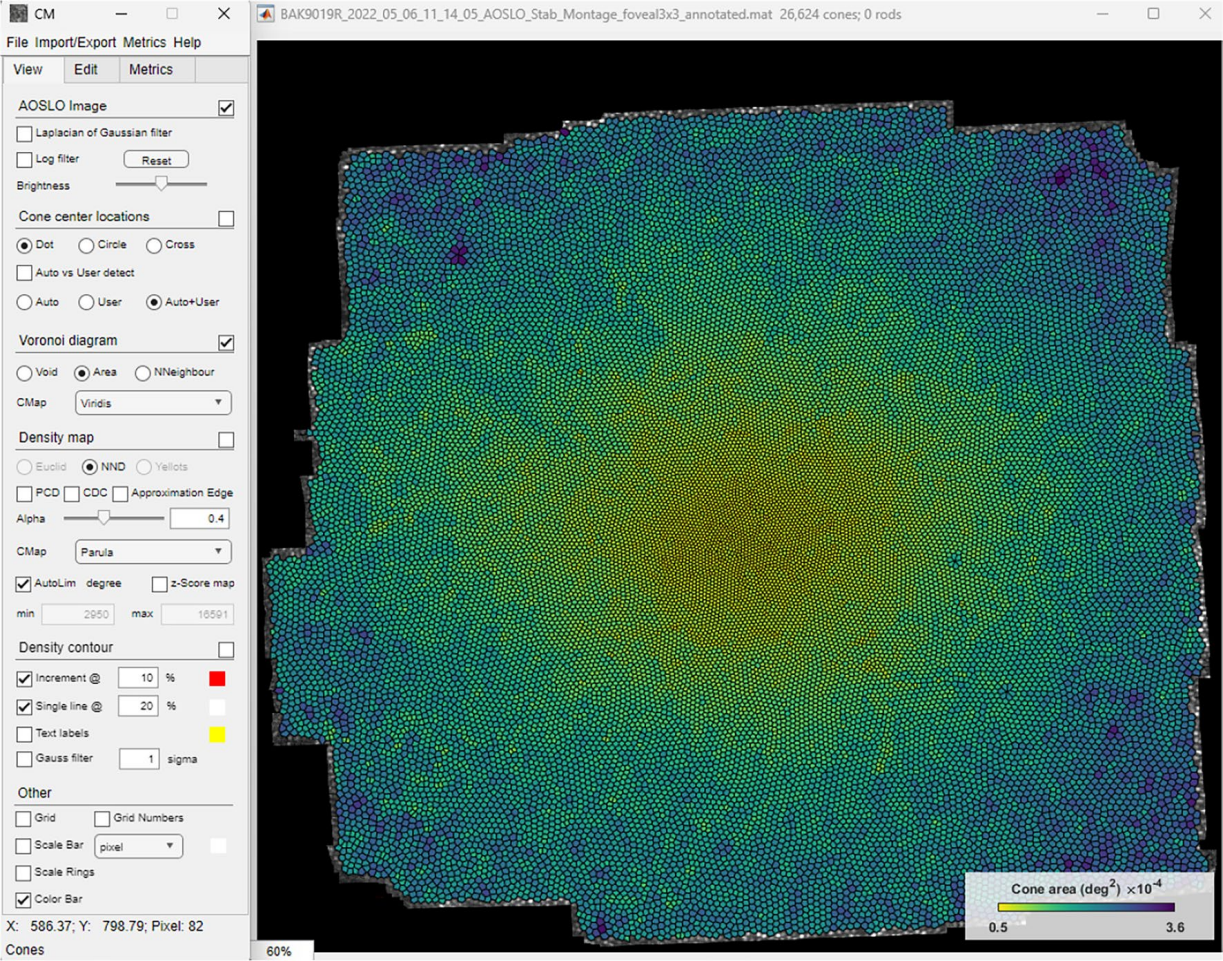
ConeMapper main interface with an example fully annotated AOSLO montage and Voronoi diagram indicating cell area.

The ConeMapper interface is made up of individual windows for maximum flexibility in window placement to accommodate different working and display environments. The main interface window from which most functions are controlled is narrow so it can be placed left or right of the image window. The image window can be freely resized, zoomed and panned with intuitive mouse controls. Digital and image scaling is displayed for repeatable settings. In general, the most important display settings are stored and carried over to future sessions (Figure 6).

**Figure 7:**
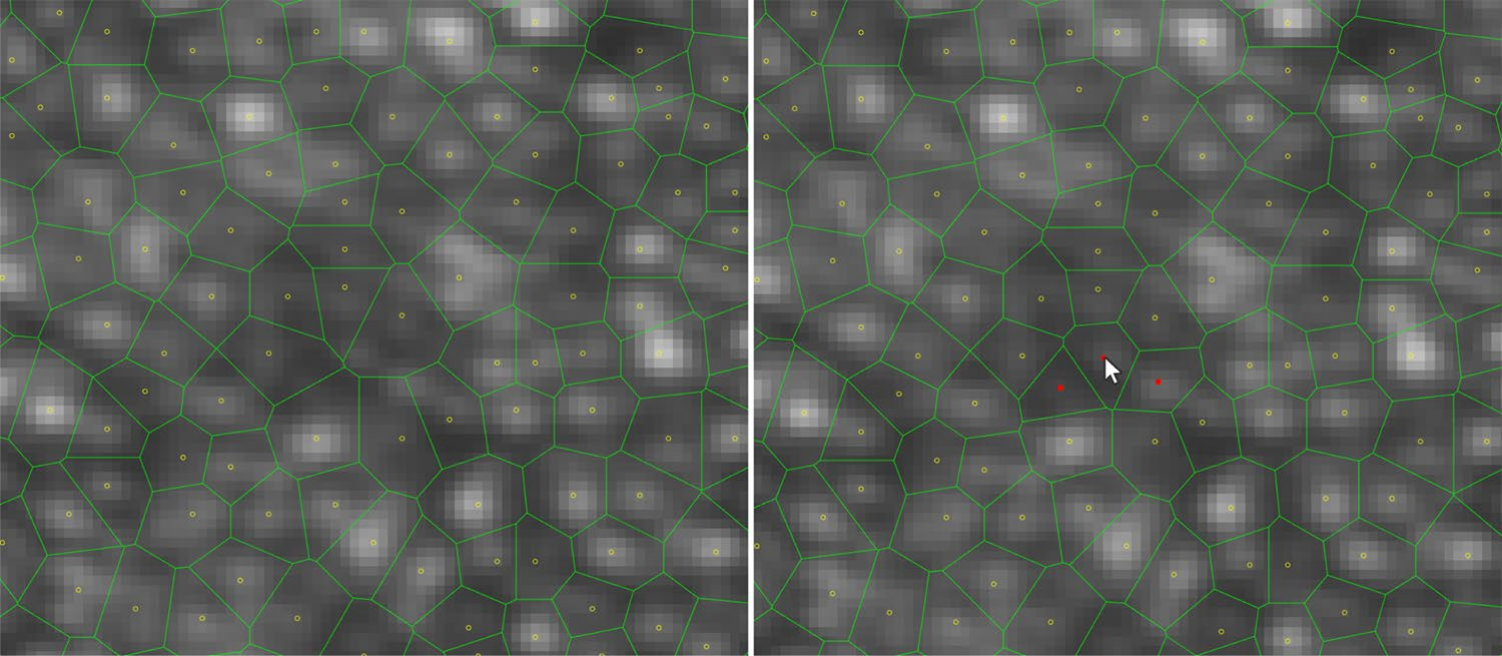
Manual annotation. The red dots in the center were manually added. In Cone Mapper by Alt+ Left Mouse Button cone is placed under the mouse position, by Alt + Right Mouse Button cone under the mouse is removed.

To activate cone annotation, the ALT-key has to be held while inputs (deletions and additions) are given with mouse clicks directly in the image window (Figure 7). Several visualizations can be employed to aid this process. What we have found to be most useful are: a grid overlay to keep track of the annotation progress, an unfilled Voronoi representation of the current cone locations which updates in real-time. For intermittent checks, a filled Voronoi cell colormap based on number of neighbors, and a z-score difference map are helpful to detect problematic areas that need re-labelling. Voronoi maps are re-generated with every change in the annotation, while the z-score map is recomputed only after computation of the associated density map. Density and density z-score maps are computed as described in the Methods section which requires a few seconds, depending on image size.

**Figure 8:**
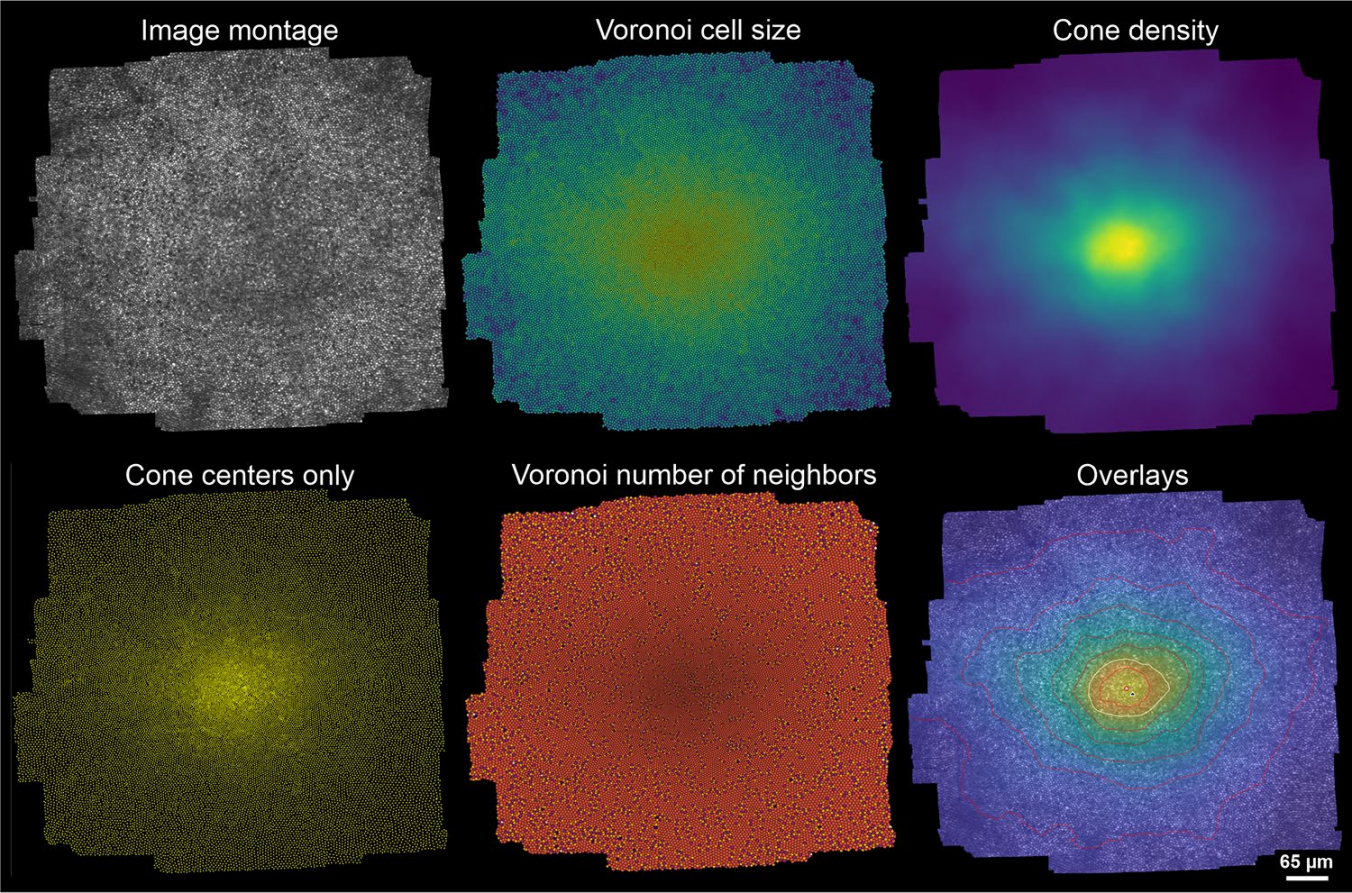
Example visualization options of ConeMapper. Visualization types are given in panel titles, colors indicate the entities given. Some or all of the individual options can be combined in layered overlays.

Most of the visualizations were implemented as layers, so they can be shown on top of each other at the same time, or in isolation (Figure 8). Other useful visualizations are markers for peak cone density (PCD) and the cone density centroid (CDC), the latter defining 0,0 eccentricity. Density contours at selectable steps can be shown to better gauge overall topography. Voronoi diagrams can be chosen to be unfilled, or filled by colormaps representing number of neighbours or area of the cell. Scale rings allows to precisely determine the distance from the CDC.

**Figure 9:**
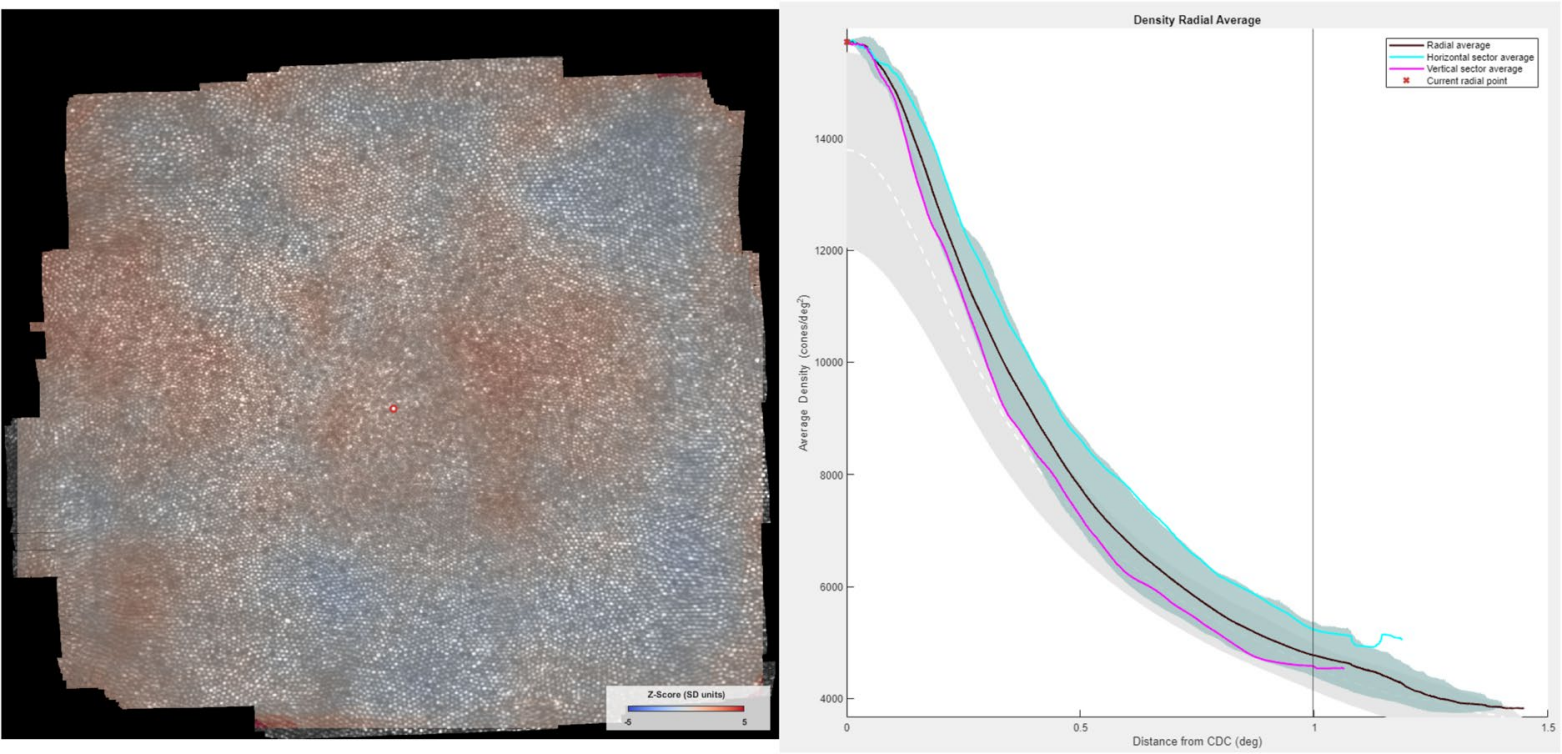
Z-Score map and Density Radial Average plot for image at figure 6. Gray zone on the background represents the mean ± 1 SD of radial density distribution.

Finally, an analysis window can be summoned to display several quantities such as key density values (CDC, PCD) and their locations, and a radial average density plot which displays the current profile in comparison to the group average. (Figure 9). Group average data can be exchanged to arbitrary datasets in the code. The user can switch between a selection of image scales and units (e.g. mm or minutes of arc) for all displayable content. For such conversions, users have to input the image magnification factor (pixel per degree of visual angle) and retinal magnification factor (micron per degree of visual angle). Such values are stored together with annotation and density maps into a single .mat file upon saving (or ending) the session.

## Discussion

We developed a software package that delivers good performance for automatic detection and manual annotation of foveolar cones and topographical analysis of en-face foveolar images. It is free to use, open source, user friendly and adjustable. Our cone recognition FCN showed improved performance when compared to published solutions, especially in the most central part of the foveola. ConeMapper allows easy manual error correction and gives instruments to immediately assess the result. Moreover, it allows extraction of basic topographical information useful for future analysis such as density profiles, two-dimensional density maps, and topographical landmarks.

Foveolar *in-vivo* image interpretation to quantify cell topography is difficult because of the blur ceiling governed by the imaging wavelength and the aberrations and numerical aperture of the eye^21^. Second, cell appearance in confocal AOSLO images is not straightforward, a consequence of the optical properties of the cells outer segments and their interaction with light^25–28^. Third, the sheer number of cells make manual annotation infeasible. Neural networks seem ideal to tackle cell detection in this instance, but require to be trained to the specific image features present in foveolar images. For example, an attempt to detect cones in our dataset with a NN that has been trained with image material from a 1-deg eccentricity region (Cunefare original data set^32^), was not able to generalize its excellent detection performance to the foveal center (Average $F_1$ score derived from our data set: 0.8452), and was en par with the inferior Fast Peak Find algorithm. Fully-automated pipelines that can handle large image eccentricities exist but lack high-confidence cone detection in the foveal center^47^.

Our improved FCN reduced manual correction times more than 2.5-fold compared to current solutions. Adding the regularity-aware particle system resulted in minor improvements to cone center localization which were outweighed by additional computation time. We thus decided to deactivate the particle system by default (it can be activated in the source code). In our experience, the remaining manual annotation error correction still takes time, but is no longer a major bottleneck in the image analysis pipeline. Other manual steps like image quality assessment, image selection and montage blending require a similar amount or more time than cone annotation.

It is important to recapitulate that successful *in-vivo* foveolar cell analysis can only be as good as the image material used. One of the key requirements for good image quality is sufficient lateral resolution. Our solution is thus tailored to work with confocal AOSLO foveal images. It has not been tested with split-detector images because this modality does not provide the resolution to resolve the small cones of the foveola^48^. Similarily, flood-illumination adaptive optics imaging does not provide the necessary lateral resolution^36^. Adaptive optics ocular coherence tomography (AO-OCT) has been shown to resolve foveolar cones and rods in select eyes^49^, but a larger image dataset is not available yet.

For future improvements to ConeMapper, we publish a roadmap of planned additions and changes alongside our software on github. These include, for example, batch processing, better figure export and ROI analysis tools. We decided to make our software and the development process open access so that there is potential for other groups to freely use and add to it. For example, ConeMapper allows choice of different NNs for automatic cell detection, which could be useful for images from outside the foveal center. For other imaging modalities such as split detection, offset pinhole, or AO-OCT imaging, specialized NNs can be added or the existing ones re-trained. In conclusion, we hope that our software package will allow researchers to speed up foveolar image analysis by making the annotation process more reliable, comfortable and comparable.

## Supplementary Information

Supplementary Information.

## References

[1] Poletti, M., Rucci, M. & Carrasco, M. Selective attention within the foveola. *Nat. Neurosci.* **20**, 1413–1417. 10.1038/nn.4622 (2017). 28805816 10.1038/nn.4622 PMC5929472

[2] Sinha, R. et al. Cellular and circuit mechanisms shaping the perceptual properties of the primate fovea. *Cell* **168**, 413-426.e12. 10.1016/j.cell.2017.01.005 (2017). 28129540 10.1016/j.cell.2017.01.005 PMC5298833

[3] Wang, Y. et al. Human foveal cone photoreceptor topography and its dependence on eye length. *Elife* **8**, e47148. 10.7554/eLife.47148 (2019). 31348002 10.7554/eLife.47148 PMC6660219

[4] Bucci, A. et al. Synchronization of visual perception within the human fovea. *Nat. Neurosc.* **43**, 127–143 (2025). 10.1038/s41593-025-02011-3 PMC12411267 40670685

[5] Yuodelis, C. & Hendrickson, A. A qualitative and quantitative analysis of the human fovea during development. *Vision. Res.* **26**, 847–855. 10.1016/0042-6989(86)90143-4 (1986). 3750868 10.1016/0042-6989(86)90143-4

[6] Cohen, M. A., Botch, T. L. & Robertson, C. E. The limits of color awareness during active, real-world vision. *Proc. Natl. Acad. Sci.* **117**, 13821–13827. 10.1073/pnas.1922294117 (2020). 32513698 10.1073/pnas.1922294117 PMC7306755

[7] Tuten, W. S. & Harmening, W. M. Foveal vision. *Curr. Biol.* **31**, R701–R703. 10.1016/j.cub.2021.03.097 (2021). 34102113 10.1016/j.cub.2021.03.097

[8] Curcio, C. A., Packer, O. & Kalina, R. E. A whole mount method for sequential analysis of photoreceptor and ganglion cell topography in a single retina. *Vision. Res.* **27**, 9–15. 10.1016/0042-6989(87)90137-4 (1987). 3303679 10.1016/0042-6989(87)90137-4

[9] Roorda, A. & Duncan, J. L. Adaptive optics ophthalmoscopy. *Annual Review of Vision Science* **1**, 19–50. 10.1146/annurev-vision-082114-035357 (2015). 26973867 10.1146/annurev-vision-082114-035357 PMC4786023

[10] Burns, S. A., Elsner, A. E., Sapoznik, K. A., Warner, R. L. & Gast, T. J. Adaptive optics imaging of the human retina. *Prog. Retin. Eye Res.* **68**, 1–30. 10.1016/j.preteyeres.2018.08.002 (2019). 30165239 10.1016/j.preteyeres.2018.08.002 PMC6347528

[11] Dubra, A. & Sulai, Y. Reflective afocal broadband adaptive optics scanning ophthalmoscope. *Biomed. Opt. Express* **2**, 1757–1768. 10.1364/BOE.2.001757 (2011). 21698035 10.1364/BOE.2.001757 PMC3114240

[12] Ameln, J., Reiniger, J. L., Hess, K., Holz, F. G. & Harmening, W. M. Supernormal foveal photoreceptor density in alport syndrome: A case report. *Eur. J. Ophthalmol.* **33**, NP51–NP54. 10.1177/11206721221093197 (2022). 35410511 10.1177/11206721221093197

[13] Kreis, J. & Carroll, J. Applications of adaptive optics imaging for studying conditions affecting the fovea. *Annual Rev. Vis. Sci.* **10**, 239–262. 10.1146/annurev-vision-102122-100022 (2024). 38635871 10.1146/annurev-vision-102122-100022

[14] Moon, B. et al. Alignment, calibration, and validation of an adaptive optics scanning laser ophthalmoscope for high-resolution human foveal imaging. *Appl. Opt.* **63**, 730–742. 10.1364/AO.504283 (2024). 38294386 10.1364/AO.504283 PMC11062499

[15] Roorda, A. Adaptive optics for studying visual function: A comprehensive review. *J. Vis.* **11**, 6. 10.1167/11.5.6 (2011). 21680646 10.1167/11.5.6 PMC6750216

[16] Tuten, W. S. et al. Spatial summation in the human fovea: Do normal optical aberrations and fixational eye movements have an effect?. *J. Vis.* **18**, 6. 10.1167/18.8.6 (2018). 30105385 10.1167/18.8.6 PMC6091889

[17] Reiniger, J. L., Domdei, N., Holz, F. G. & Harmening, W. M. Human gaze is systematically offset from the center of cone topography. *Curr. Biol.* **31**, 4188-4193.e3. 10.1016/j.cub.2021.07.005 (2021). 34343479 10.1016/j.cub.2021.07.005

[18] Witten, J. L., Lukyanova, V. & Harmening, W. M. Sub-cone visual resolution by active, adaptive sampling in the human foveola. *Elife* **13**, RP98648. 10.7554/eLife.98648 (2024). 39468921 10.7554/eLife.98648 PMC11521370

[19] Yamada, E. Some Structural Features of the Fovea Centralis in the Human Retina. *Arch. Ophthalmol.* **82**, 151–159. 10.1001/archopht.1969.00990020153002 (1969). 4183671 10.1001/archopht.1969.00990020153002

[20] Domdei, N. et al. Cone density is correlated to outer segment length and retinal thickness in the human foveola. *Invest. Ophthalmol. Vis. Sci.* **64**, 11. 10.1167/iovs.64.15.11 (2023). 38064229 10.1167/iovs.64.15.11 PMC10709802

[21] Campbell, F. W. & Green, D. G. Optical and retinal factors affecting visual resolution. *J. Physiol.* **181**, 576–593. 10.1113/jphysiol.1965.sp007784 (1965). 5880378 10.1113/jphysiol.1965.sp007784 PMC1357668

[22] Curcio, C. A. & Sloan, K. R. Packing geometry of human cone photoreceptors: Variation with eccentricity and evidence for local anisotropy. *Vis. Neurosci.* **9**, 169–180. 10.1017/S0952523800009639 (1992). 1504026 10.1017/s0952523800009639

[23] Pum, D., Ahnelt, P. K. & Grasl, M. Iso-orientation areas in the foveal cone mosaic. *Vis. Neurosci.* **5**, 511–523. 10.1017/S0952523800000687 (1990). 2085468 10.1017/s0952523800000687

[24] Putnam, N. M., Hammer, D. X., Zhang, Y., Merino, D. & Roorda, A. Modeling the foveal cone mosaic imaged with adaptive optics scanning laser ophthalmoscopy. *Opt. Express* **18**, 24902–24916. 10.1364/OE.18.024902 (2010). 21164835 10.1364/OE.18.024902 PMC3408900

[25] Pallikaris, A., Williams, D. R. & Hofer, H. The reflectance of single cones in the living human eye. *Invest. Ophthalmol. Vis. Sci.* **44**, 4580–4592. 10.1167/iovs.03-0094 (2003). 14507907 10.1167/iovs.03-0094

[26] Bruce, K. S. et al. Normal perceptual sensitivity arising from weakly reflective cone photoreceptors. *Invest. Ophthalmol. Vis. Sci.* **56**, 4431–4438. 10.1167/iovs.15-16547 (2015). 26193919 10.1167/iovs.15-16547 PMC4509056

[27] Meadway, A. & Sincich, L. C. Light reflectivity and interference in cone photoreceptors. *Biomed. Opt. Express* **10**, 6531–6554. 10.1364/BOE.10.006531 (2019). 31853415 10.1364/BOE.10.006531 PMC6913404

[28] Bensinger, E., Wang, Y. & Roorda, A. Patches of dysflective cones in eyes with no known disease. *Invest. Ophthalmol. Vis. Sci.* **63**, 29–29. 10.1167/iovs.63.1.29 (2022). 35072690 10.1167/iovs.63.1.29 PMC8802026

[29] Xue, B., Choi, S. S., Doble, N. & Werner, J. S. Photoreceptor counting and montaging of en-face retinal images from an adaptive optics fundus camera. *J. Opt. Soc. Am. A* **24**, 1364–1372. 10.1364/JOSAA.24.001364 (2007). 10.1364/josaa.24.001364 PMC2583217 17429482

[30] Turpin, A., Morrow, P., Scotney, B., Anderson, R. & Wolsley, C. Automated identification of photoreceptor cones using multi-scale modelling and normalized cross-correlation. In *Image Analysis and Processing - ICIAP 2011, 494–503 (Springer* (eds Maino, G. & Foresti, G. L.) (Berlin Heidelberg, Berlin, Heidelberg, 2011).

[31] Cooper, R. F., Aguirre, G. K. & Morgan, J. I. W. Fully automated estimation of spacing and density for retinal mosaics. *Translational Vision Science & Technology* **8**, 26. 10.1167/tvst.8.5.26 (2019). 10.1167/tvst.8.5.26 PMC6798313 31637106

[32] Cunefare, D. et al. Open source software for automatic detection of cone photoreceptors in adaptive optics ophthalmoscopy using convolutional neural networks. *Sci. Rep.* **7**, 6620. 10.1038/s41598-017-07103-0 (2017). 28747737 10.1038/s41598-017-07103-0 PMC5529414

[33] Davidson, B. et al. Automatic cone photoreceptor localisation in healthy and stargardt afflicted retinas using deep learning. *Sci. Rep.* **8**, 7911. 10.1038/s41598-018-26350-3 (2018). 29784939 10.1038/s41598-018-26350-3 PMC5962538

[34] Hamwood, J., Alonso-Caneiro, D., Sampson, D. M., Collins, M. J. & Chen, F. K. Automatic detection of cone photoreceptors with fully convolutional networks. *Translational Vision Science & Technology* **8**, 10. 10.1167/tvst.8.6.10 (2019). 10.1167/tvst.8.6.10 PMC6855369 31737434

[35] Li, K., Yin, Q., Ren, J., Song, H. & Zhang, J. Automatic quantification of cone photoreceptors in adaptive optics scanning light ophthalmoscope images using multi-task learning. *Biomed. Opt. Express* **13**, 5187–5201. 10.1364/BOE.471426 (2022). 36425624 10.1364/BOE.471426 PMC9664876

[36] Wooning, S. et al. Automated cone photoreceptor detection in adaptive optics flood illumination ophthalmoscopy. *Ophthalmology Science* **5**, 100675. 10.1016/j.xops.2024.100675 (2025). 40114708 10.1016/j.xops.2024.100675 PMC11925573

[37] Cava, J. A. et al. Assessing interocular symmetry of the foveal cone mosaic. *Investigative Ophthalmology & Visual Science* **61**, 23. 10.1167/iovs.61.14.23 (2020). 10.1167/iovs.61.14.23 PMC7746960 33331861

[38] Morgan, J. I. W., Chen, M., Huang, A. M., Jiang, Y. Y. & Cooper, R. F. Cone identification in choroideremia: Repeatability, reliability, and automation through use of a convolutional neural network. *Translational Vision Science & Technology* **9**, 40. 10.1167/tvst.9.2.40 (2020). 10.1167/tvst.9.2.40 PMC7424931 32855844

[39] Wynne, N. et al. Intergrader agreement of foveal cone topography measured using adaptive optics scanning light ophthalmoscopy. *Biomed. Opt. Express* **13**, 4445–4454. 10.1364/BOE.460821 (2022). 36032569 10.1364/BOE.460821 PMC9408252

[40] Ameln, J. et al. In-vivo cone photoreceptor topography of the normal human foveola. *Investigative Ophthalmology & Visual Science* **64**, 1040 (2023). 10.1167/iovs.66.11.13 PMC12347159 40767443

[41] Domdei, N. et al. Ultra-high contrast retinal display system for single photoreceptor psychophysics. *Biomed. Opt. Express* **9**, 157–172. 10.1364/BOE.9.000157 (2018). 29359094 10.1364/BOE.9.000157 PMC5772572

[42] Domdei, N., Linden, M., Reiniger, J. L., Holz, F. G. & Harmening, W. M. Eye tracking-based estimation and compensation of chromatic offsets for multi-wavelength retinal microstimulation with foveal cone precision. *Biomed. Opt. Express* **10**, 4126–4141. 10.1364/BOE.10.004126 (2019). 31452999 10.1364/BOE.10.004126 PMC6701545

[43] Stevenson, S. B., Roorda, A. & Kumar, G. Eye tracking with the adaptive optics scanning laser ophthalmoscope. In *Proceedings of the 2010 Symposium on Eye-Tracking Research & Applications*, ETRA ’10, 195–198, 10.1145/1743666.1743714(Association for Computing Machinery, New York, NY, USA, 2010).

[44] Chen, M. et al. Multi-modal automatic montaging of adaptive optics retinal images. *Biomed. Opt. Express* **7**, 4899–4918. 10.1364/BOE.7.004899 (2016). 28018714 10.1364/BOE.7.004899 PMC5175540

[45] Ronneberger, O., Fischer, P. & Brox, T. U-net: Convolutional networks for biomedical image segmentation. In Navab, N., Hornegger, J., Wells, W. M. & Frangi, A. F. (eds.) *Medical Image Computing and Computer-Assisted Intervention – MICCAI 2015*, 234–241 (Springer International Publishing, Cham, 2015).

[46] K. He, X. Zhang, S. Ren & J. Sun. Deep residual learning for image recognition. In *2016 IEEE Conference on Computer Vision and Pattern Recognition (CVPR)*, 770–778, 10.1109/CVPR.2016.90(2016).

[47] Natan, A. "Fast 2D peak finder". MATLAB Central File Exchange. Available at https://www.mathworks.com/matlabcentral/fileexchange/37388-fast-2d-peak-finder (2021).

[48] Cooper, R. F., Kalaparambath, S., Aguirre, G. K. & Morgan, J. I. W. Morphology of the normative human cone photoreceptor mosaic and a publicly available adaptive optics montage repository. *Sci. Rep.* **14**, 23166. 10.1038/s41598-024-74274-y (2024). 39369063 10.1038/s41598-024-74274-y PMC11455974

[49] Scoles, D. et al. In vivo imaging of human cone photoreceptor inner segments. *Investigative Ophthalmology & Visual Science* **55**, 4244–4251. 10.1167/iovs.14-14542 (2014). 24906859 10.1167/iovs.14-14542 PMC4095721

[50] Felberer, F. et al. Adaptive optics slo/oct for 3d imaging of human photoreceptors in vivo. *Biomed. Opt. Express* **5**, 439–456. 10.1364/BOE.5.000439 (2014). 24575339 10.1364/BOE.5.000439 PMC3920875

