# Correction: Neural network assisted annotation and analysis tool to study in-vivo foveolar cone photoreceptor topography

**DOI:** 10.1038/s41598-025-16172-5

**Published:** 2025-08-29

**Authors:** Aleksandr Gutnikov, Patrick Hähn-Schumacher, Julius Ameln, Shekoufeh Gorgi Zadeh, Thomas Schultz, Wolf Harmening

**Affiliations:** 1 Department of Ophthalmology, University Hospital Bonn, Bonn, 53127 Germany; 2 b-it and Computer Science Department, University of Bonn, Bonn, 53115 Germany; 3 Lamarr Institute for Machine Learning and Artificial Intelligence,; 4 Present Address: Luxembourg Centre for Systems Biomedicine, University of Luxembourg, Belvaux, Luxembourg

Correction to: *Scientific Reports* 10.1038/s41598-025-08028-9, Published: 04 July 2025

In the original article, the authors omitted the below Reference, which is listed below as Reference 47.

47. Natan, A. “Fast 2D peak finder”. MATLAB Central File Exchange. Available at https://www.mathworks.com/matlabcentral/fileexchange/37388-fast-2d-peak-finder (2021).

As a result, in the section ‘Automatic foveolar cone detection’,

“To evaluate automatic annotation performance of our software, key performance metrics were computed for a validation data set and compared to the performance of two published neural networks (Hamwood FCN^34^, Cunefare CNN^32^), and to a conventional fast peak find algorithm (FPF, Fast 2d Peak Finder MATLAB File Exchange) on the same set.”

now reads:

“To evaluate automatic annotation performance of our software, key performance metrics were computed for a validation data set and compared to the performance of two published neural networks (Hamwood FCN^34^, Cunefare CNN^32^), and to a conventional fast peak find algorithm (FPF, Fast 2d Peak Finder MATLAB File Exchange^47^,) on the same set.”

As a result of the changes, the References have been renumbered.

The original Article has been corrected.

