# Supplementary material for: Neural network assisted annotation and analysis tool to study in-vivo foveolar cone photoreceptor topography
Source: Sci Rep. 2025 Jul 4;15:23858. doi: 10.1038/s41598-025-08028-9 (PMC12227681; doi:10.1038/s41598-025-08028-9)
Supplement: Supplementary file 1 — Supplementary Information. [file 41598_2025_8028_MOESM1_ESM.pdf]

Neural network assisted annotation and analysis tool to study *in-vivo* foveolar cone photoreceptor topography

Aleksandr Gutnikov[1,+], Patrick Hähn-Schumacher[2,+], Julius Ameln[1], Shekoufeh Gorgi Zadeh[2, †], Thomas Schultz[2, 3], and Wolf Harmening[1,*]

[1]Department of Ophthalmology, University Hospital Bonn, 53127, Germany
[2]b-it and Computer Science Department, University of Bonn, 53115, Germany
[3]Lamarr Institute for Machine Learning and Artificial Intelligence, https://lamarr-institute.org/
[*]Corresponding author: wolf.harmening@ukbonn.de
[+]these authors contributed equally to this work
[†]current affiliation: Luxembourg Centre for Systems Biomedicine, University of Luxembourg, Belvaux, Luxembourg

Supplementary information

Table S1. Individual true positive rates (TPR) for all montages in the validation set. Higher values indicate better performance.

Subject	FastPeakFinder	Cunefare	Hamwood	Our FCN
BAK1088L	0.9085	0.8679	0.8294	0.9226
BAK1088R	0.9215	0.8897	0.8487	0.9370
BAK1089L	0.9582	0.9575	0.9250	0.9877
BAK1089R	0.9643	0.9585	0.9283	0.9886
BAK1093L	0.9676	0.9767	0.9559	0.9833
BAK1093R	0.9513	0.9769	0.9568	0.9783
BAK1101L	0.9667	0.9711	0.9552	0.9922
BAK1101R	0.9734	0.9711	0.9625	0.9919
BAK1102L	0.9632	0.9601	0.9262	0.9903
BAK1102R	0.9562	0.9374	0.9168	0.9840
BAK8001L	0.9546	0.9657	0.9237	0.9889
BAK8001R	0.9515	0.9480	0.9082	0.9884
Average	0.9531	0.9484	0.9197	0.9778
STD	0.0192	0.0348	0.0418	0.0229

Table S2. Individual false detection rate (FDR) for all montages in the validation set. Lower values indicate better performance.

Subject	FastPeakFinder	Cunefare	Hamwood	Our FCN
BAK1088L	0.1590	0.0674	0.0522	0.0447
BAK1088R	0.1784	0.0588	0.0442	0.0372
BAK1089L	0.2283	0.0458	0.0261	0.0201
BAK1089R	0.2168	0.0400	0.0222	0.0160
BAK1093L	0.2397	0.0318	0.0196	0.0234
BAK1093R	0.2438	0.0335	0.0233	0.0297
BAK1101L	0.1448	0.0354	0.0215	0.0185
BAK1101R	0.1109	0.0308	0.0193	0.0162
BAK1102L	0.1554	0.0417	0.0249	0.0205
BAK1102R	0.1797	0.0478	0.0297	0.0220
BAK8001L	0.2414	0.0449	0.0243	0.0186
BAK8001R	0.2367	0.0583	0.0265	0.0189
Average	0.1946	0.0447	0.0278	0.0238
STD	0.0456	0.0117	0.0101	0.0089

Table S3. Individual F_1 score for all montages in the validation set. Higher values indicate better performance.

Subject	FastPeakFinder	Cunefare	Hamwood	Our FCN
BAK1088L	0.8734	0.8991	0.8847	0.9387
BAK1088R	0.8687	0.9147	0.8991	0.9497
BAK1089L	0.8549	0.9559	0.9488	0.9838
BAK1089R	0.8644	0.9592	0.9524	0.9863
BAK1093L	0.8515	0.9724	0.9680	0.9799
BAK1093R	0.8426	0.9717	0.9666	0.9743
BAK1101L	0.9075	0.9679	0.9667	0.9868
BAK1101R	0.9293	0.9702	0.9715	0.9878
BAK1102L	0.9001	0.9592	0.9500	0.9849
BAK1102R	0.8830	0.9448	0.9428	0.9810
BAK8001L	0.8454	0.9604	0.9490	0.9852
BAK8001R	0.8471	0.9448	0.9397	0.9848
Average	0.8723	0.9517	0.9449	0.9769
STD	0.0277	0.0231	0.0271	0.0159

Table S4. Individual true positive rates (TPR) for all montages in the validation set. Radius of the area is 0.3 deg with the center in ground truth CDC point. Higher values indicate better performance.

Subject	FastPeakFinder	Cunefare	Hamwood	Our FCN
BAK1088L	0.9820	0.9925	0.9856	0.9991
BAK1088R	0.9750	0.9879	0.9726	0.9951
BAK1089L	0.9483	0.9817	0.9666	0.9956
BAK1089R	0.9572	0.9816	0.9708	0.9971
BAK1093L	0.9467	0.9798	0.9683	0.9791
BAK1093R	0.9352	0.9720	0.9675	0.9783
BAK1101L	0.9638	0.9926	0.9846	0.9975
BAK1101R	0.9878	0.9950	0.9845	0.9982
BAK1102L	0.9660	0.9893	0.9600	0.9962
BAK1102R	0.9743	0.9870	0.9536	0.9958
BAK8001L	0.9495	0.9898	0.9700	0.9977
BAK8001R	0.9313	0.9688	0.9326	0.9880
Average	0.9597	0.9848	0.9681	0.9931
STD	0.0181	0.0083	0.0148	0.0073

Table S5. Individual false detection rate (FDR) for all montages in the validation set. Radius of the area is 0.3 deg with the center in ground truth CDC point. Lower values indicate better performance.

Subject	FastPeakFinder	Cunefare	Hamwood	Our FCN
BAK1088L	0.3390	0.0075	0.0016	0.0016
BAK1088R	0.4302	0.0224	0.0081	0.0078
BAK1089L	0.5466	0.0380	0.0079	0.0084
BAK1089R	0.5595	0.0365	0.0072	0.0059
BAK1093L	0.5858	0.0440	0.0128	0.0184
BAK1093R	0.5873	0.0524	0.0180	0.0297
BAK1101L	0.3987	0.0110	0.0040	0.0040
BAK1101R	0.2661	0.0029	0.0015	0.0018
BAK1102L	0.4115	0.0202	0.0057	0.0082
BAK1102R	0.4280	0.0188	0.0045	0.0064
BAK8001L	0.5182	0.0290	0.0069	0.0059
BAK8001R	0.5467	0.0639	0.0176	0.0229
Average	0.4681	0.0289	0.0080	0.0101
STD	0.1044	0.0186	0.0055	0.0088

Table S6. Individual F_1 scores for all montages in the validation set. Radius of the area is 0.3 deg with the center in ground truth CDC point. Higher values indicate better performance.

Subject	FastPeakFinder	Cunefare	Hamwood	Our FCN
BAK1088L	0.7901	0.9925	0.9920	0.9988
BAK1088R	0.7192	0.9827	0.9822	0.9937
BAK1089L	0.6135	0.9717	0.9792	0.9936
BAK1089R	0.6033	0.9724	0.9817	0.9956
BAK1093L	0.5762	0.9677	0.9777	0.9804
BAK1093R	0.5727	0.9597	0.9747	0.9743
BAK1101L	0.7406	0.9908	0.9903	0.9967
BAK1101R	0.8421	0.9961	0.9915	0.9982
BAK1102L	0.7314	0.9845	0.9768	0.9940
BAK1102R	0.7208	0.9841	0.9741	0.9947
BAK8001L	0.6392	0.9803	0.9814	0.9959
BAK8001R	0.6098	0.9521	0.9569	0.9825
Average	0.6799	0.9779	0.9799	0.9915
STD	0.0890	0.0134	0.0096	0.0079

Table S7. The estimate time needed for an experienced user to correct the errors of the algorithm in minutes. This estimate assumes that it takes 3 seconds to correct one error (FP+FN). It is thus very approximate. Actual correction times highly depend on eccentricity, image quality and the presence of rods in the region of interest.

Subject	FastPeakFinder	Cunefare	Hamwood	Our FCN
BAK1088L	351	301	355	179
BAK1088R	326	231	282	134
BAK1089L	386	130	155	48
BAK1089R	383	127	152	43
BAK1093L	428	88	105	64
BAK1093R	455	89	106	81
BAK1101L	234	86	90	35
BAK1101R	172	76	73	31
BAK1102L	247	107	135	40
BAK1102R	272	139	147	47
BAK8001L	351	100	133	37
BAK8001R	369	147	167	41
Median	350	117	141	45
